# Specific role of RhoC in tumor invasion and metastasis

**DOI:** 10.18632/oncotarget.20957

**Published:** 2017-09-16

**Authors:** Sarah Lang, Hauke Busch, Melanie Boerries, Tilman Brummer, Sylvia Timme, Silke Lassmann, Klaus Aktories, Gudula Schmidt

**Affiliations:** ^1^ Institute for Experimental and Clinical Pharmacology and Toxicology, Faculty of Medicine, Albert-Ludwigs-University, Freiburg, Germany; ^2^ Lübeck Institute of Experimental Dermatology, Institute for Cardiogenetics, University of Lübeck, Lübeck, Germany; ^3^ Institute of Molecular Medicine and Cell Research, Faculty of Medicine, University of Freiburg, Freiburg, Germany; ^4^ German Cancer Consortium (DKTK), Freiburg, Germany, German Cancer Research Center (DKFZ), Heidelberg, Germany; ^5^ Center for Biological Signalling Studies (BIOSS), University of Freiburg, Freiburg, Germany; ^6^ Institute for Surgical Pathology, Medical Center, Faculty of Medicine, University of Freiburg, Freiburg, Germany

**Keywords:** Rho GTPase, invasion, cyclooxygenase, breast cancer

## Abstract

Rho GTPases are regulators of many cellular functions and are often dysregulated in cancer. However, the precise role of Rho proteins for tumor development is not well understood. In breast cancer, overexpression of RhoC is linked with poor prognosis. Here, we aim to compare the function of RhoC and its homolog family member RhoA in breast cancer progression. We established stable breast epithelial cell lines with inducible expression of RhoA and RhoC, respectively. Moreover, we made use of Rho-activating bacterial toxins (Cytotoxic Necrotizing Factors) to stimulate the endogenous pool of Rho GTPases in benign breast epithelial cells and simultaneously knocked down specific Rho proteins. Whereas activation of Rho GTPases was sufficient to induce an invasive phenotype in three-dimensional culture systems, overexpression of RhoA or RhoC were not. However, RhoC but not RhoA was required for invasion, whereas RhoA and RhoC equally regulated proliferation. We further identified downstream target genes of RhoC involved in invasion and identified PTGS2 (COX-2) being preferentially upregulated by RhoC. Consistently, the COX-2 inhibitor Celecoxib blocked the invasive phenotype induced by the Rho-activating toxins.

## INTRODUCTION

Rho GTPases belong to the Ras superfamily of small GTPases. They are molecular switches cycling between a GDP-bound off- and a GTP-bound on-state. Activation occurs via nucleotide exchange, which is catalyzed by guanine nucleotide exchange factors (GEFs). Inactivation is achieved by hydrolysis of the bound GTP to GDP. This is stimulated by GTPase-activating proteins (GAPs). Rho proteins are master regulators of the cytoskeleton and are therefore essentially involved in cell migration, adhesion and polarity. Moreover, they are key molecules controlling further cellular functions like gene transcription and proliferation [1]. Recent studies have shown that dysregulation of Rho GTPases plays a pivotal role in human tumor development [2]. However, the precise role of Rho proteins in these processes is not well understood. Particularly the different functions of RhoA and RhoC, which mainly interact with the same spectrum of effector molecules, remains enigmatic. However, differences in localization and distinct affinities to effectors have been reported [3, 4].

A growing number of reports focus on RhoC as an essential factor for invasion and metastasis of various types of tumor cells [5–9], whereas RhoA rather seems to play a role for proliferation instead [10–12]. In colorectal cancer, however, RhoA seems to have low impact on proliferation but is essential for invasion and migration [13]. Only few studies directly compared the ability of the two closely related Rho GTPases to induce proliferation and invasion [14, 15]. RhoC overexpression was especially linked to aggressive cancers as for example inflammatory breast cancer, which metastasizes rapidly [16, 17]. In breast epithelia, progression from a persistent to an invasive phenotype requires loss of epithelial polarity and deregulation of cellular adhesion. This epithelial-mesenchymal transition (EMT) includes a change in gene expression patterns, which can be induced by several transcription factors, like Snail, ZEB1 or Twist [18–20]. Rho GTPases directly regulate adhesion and polarity of epithelial cells [21]. As RhoC is frequently upregulated in inflammatory breast cancer [22], we intended to analyze its potential for inducing EMT, migration and invasion and to regulate specific genes involved in tumorigenesis. Unlike for Ras, no typical activating mutant of RhoC was detected in human tumors so far, but diverse missense mutations have been identified with low frequency in several cancer types (cbioportal.org). Different from Ras, permanent GTP-loading of Rho GTPases may even be a disadvantage for tumorigenesis, since constitutive Rho activation has been reported to block rather than to stimulate cell migration [23–25].

To obtain more insight into the role of RhoA and RhoC in the development of human breast cancer, we established stable cell lines whose expression of RhoA or RhoC can be induced reversibly. We additionally made use of two bacterial toxins: *Escherichia coli* Cytotoxic Necrotizing Factor 1 (CNF1) and *Yersinia pseudotuberculosis* Cytotoxic Necrotizing Factor (CNFY) to directly activate the endogenous pool of Rho GTPases. Both toxins enter mammalian cells by receptor-mediated endocytosis and are released from the endosome into the cytosol. They catalyze the deamidation of Rho GTPases at a crucial glutamine to generate glutamic acid. Because this glutamine is essential for GTP hydrolysis, its aforementioned modification leads to the block of the inactivation step in the GTPase cycle and therefore to permanent activation of the molecular switches [26, 27]. Whereas CNF1 deamidates a broad range of Rho GTPases including Rac1 and CDC42, the highly preferred substrates of CNFY are RhoA, B, and C [28].

As a cellular system, we used MCF-10A cells, which are non-transformed mammary epithelial cells capable in forming organoid-like acinar structures in three-dimensional culture systems [29, 30]. They are widely used to characterize the impact of expressed proteins on epithelial morphogenesis, growth factor dependence, apico-basal polarity and luminal cell death. Therefore, MCF-10A cells constitute an ideal model system to unravel the contribution of Rho GTPases during the transition to breast carcinoma. We characterized changes in morphology, migration and invasion upon induction of RhoA/RhoC expression or activation by toxins in two- and three-dimensional systems. By comparative microarray analysis, we identified RhoC-specific target genes involved in pro-migratory changes.

## RESULTS

### RhoC expression in human ductal breast cancer

Data from the literature indicate that RhoC expression may occur at selective stages of tumor progression and metastasis [22]. Based on these assumptions, we analyzed RhoC protein expression as well as RhoA protein expression in human tissue specimens of matched normal epithelium, ductal carcinoma *in situ* (DCIS) and ductal breast carcinomas of 9 cases (with 1/9 cases pTis, pNx; 8/9 cases with pT1b to pT2 and pN0 to pN2a) by immunohistochemistry.

**Figure 1:**
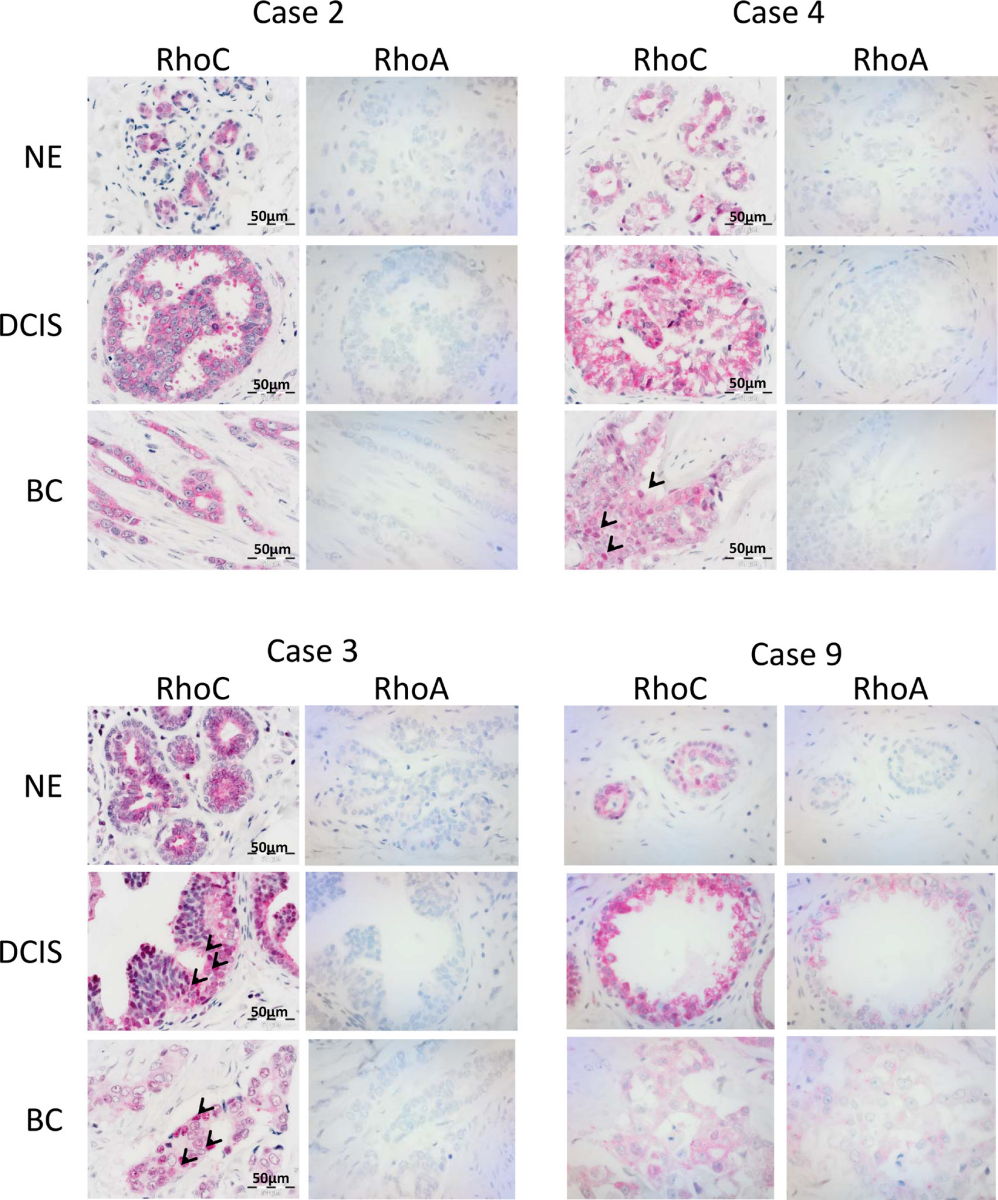
RhoC is expressed in ductal breast carcinomas as well as precursor lesions and non-neoplastic epithelia The figure shows selected cases with immunohistochemical analysis of RhoC and RhoA protein expression in matched tissue specimens of non-neoplastic epithelium (NE), ductal carcinoma *in situ* (DCIS) and ductal breast carcinoma (BC). Note cytoplasmic positivity of RhoC in all specimens and additional nuclear positivity in BC of case #4 and in DCIS and BC of case #3 (examples of cells marked by arrows).

As shown in Figure 1, RhoC and RhoA showed different patterns of expression, thereby also underlying the specificity of the immunohistochemical stainings. RhoC was expressed in all evaluated matched normal epithelia, DCIS and ductal breast carcinomas. Of note, its expression was mainly cytoplasmic, but in part also nuclear (Supplementary Table 1). In contrast, an absent RhoA staining was found in most cases and tissue specimens of normal epithelium, DCIS and ductal breast carcinomas, except for three out of nine cases showing weak cytoplasmic RhoA protein expression in DCIS and/or ductal breast carcinomas (Figure 1, case 9). The data indicate that RhoC may be more important for breast cancer development compared to RhoA.

### Activation of Rho GTPases by bacterial toxins is sufficient to induce an invasive phenotype of MCF-10A breast epithelial cells

**Figure 2:**
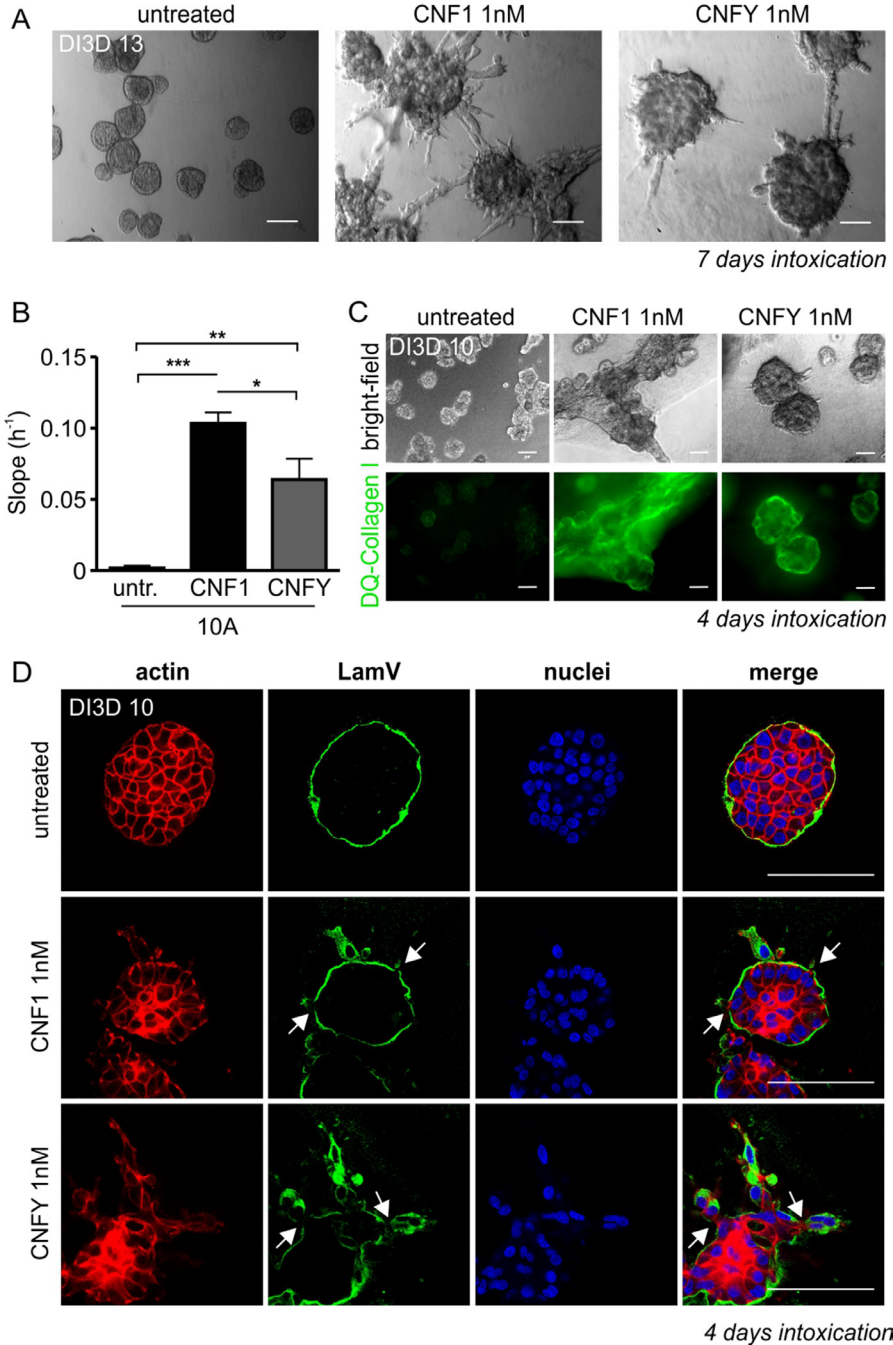
Intoxication with the Rho-activating bacterial toxins CNF1 and CNFY induces an invasive phenotype in three-dimensional (3D) MCF-10A cultures (**A**) Representative bright-field micrographs of wild type (WT-) MCF-10A acini grown in 3D Matrigel/Collagen I (1:1) overlay cultures (days in 3D; DI3D 13). After 6 days in culture, cells were intoxicated for 7 days with CNF1 (middle) or CNFY (right) and compared to untreated control cells (left); scale bar 50 μm. (**B**) Quantification of the functional invasiveness of MCF-10A cells using the Xcelligence^®^ system. Cells were treated with 1 nM CNF1 (10A+1, black) and 1 nM CNFY (10A+Y, grey) after cell settlement and before beginning of the measurement. (**C**) Top, bright-field micrographs showing untreated (left), CNF1- (middle) and CNFY-intoxicated (right) WT-MCF-10A 3D cultures (images taken at DI3D 10, intoxication during DI3D 7-10). Bottom: corresponding epifluorescent images of DQ-Collagen I, scale bar 50 μm. (**D**) Confocal images of single MCF-10A acini (DI3D 13, control (top), CNF1-treated (middle) and CNFY-intoxicated (bottom; DI3D 7-13)) stained for f-actin (red) and the basement membrane component Laminin V, (green); scale bar 50 μm. ^*^p < 0.05; ^**^p < 0.01; ^***^p < 0.001; multiple comparison ANOVA.

MCF-10A cells are spontaneously immortalized, non-transformed mammary epithelial cells. They form spherical, organoid-like acinar structures in Matrigel/Collagen I. Interestingly, stimulation of Rho GTPases by the bacterial toxins CNF1 or CNFY in pre-formed acini stimulated the formation of protrusions and invasive strands (Figure 2A). CNF1 showed a stronger effect compared to CNFY. This can be explained by the fact that CNF1 activates a broader spectrum of Rho GTPases compared to CNFY, upon which invasive outgrowth is further sustained. Similarly, both toxins enhanced single cell invasion into the extracellular matrix as determined by an impedance-measured invasion assay (Figure 2B). Invasive processes frequently include the activation of matrix metalloproteases. This was studied by cultivating MCF-10A cells in DQ-Collagen I, which exclusively emits a fluorescent signal following its degradation (Figure 2C). Moreover, toxin treatment induced local destruction of the basement membrane built around acini as shown by disrupted Laminin V staining (Figure 2D). Taken together, Rho protein activation was sufficient to induce all properties required for an invasive phenotype. Pulldown experiments showed that the toxins prominently activated RhoA and RhoC in MCF-10A cells about 3- and 7-fold in case of CNF1 and CNFY, respectively (Supplementary Figure 1). Expression of these two Rho proteins is dysregulated in breast cancer tissues: RhoC is overexpressed in 0.5% of all cases and downregulated in 0.2% of cases (4525 tissues analyzed) whereas RhoA is more frequently downregulated (with 0.1% higher expression and 0.5% downregulation; http://www.cbioportal.org). We intended to analyze and compare the specific role of RhoA and RhoC in inducing the invasive phenotype of MCF-10A cells.

### Generation and characterization of MCF-10A cell lines

To unravel the contribution of RhoA and RhoC during the development of non-transformed breast epithelial cells to invasive carcinoma, we established stable MCF-10A cell lines, in which expression of RhoA or RhoC can be induced by doxycycline. We generated bi-cistronic constructs allowing expression of RhoA/RhoC and green fluorescent protein (GFP). Gene expression was set under the control of a doxycycline-regulated trans-activator and a transcriptional silencer for conditional expression of RhoA and RhoC, respectively [29]. First, we characterized the cell lines by analyzing time and concentration dependency of doxycycline induction and studied stability of the induced protein expression by washout experiments (Supplementary Figure 2). Based on these experiments we chose 2 μg/ml doxycycline to induce expression of Rho proteins. Overexpression of the proteins was detectable for ~48 h following washout of the antibiotic.

### Overexpressionof RhoA or RhoC is not sufficient to induce invasion

**Figure 3:**
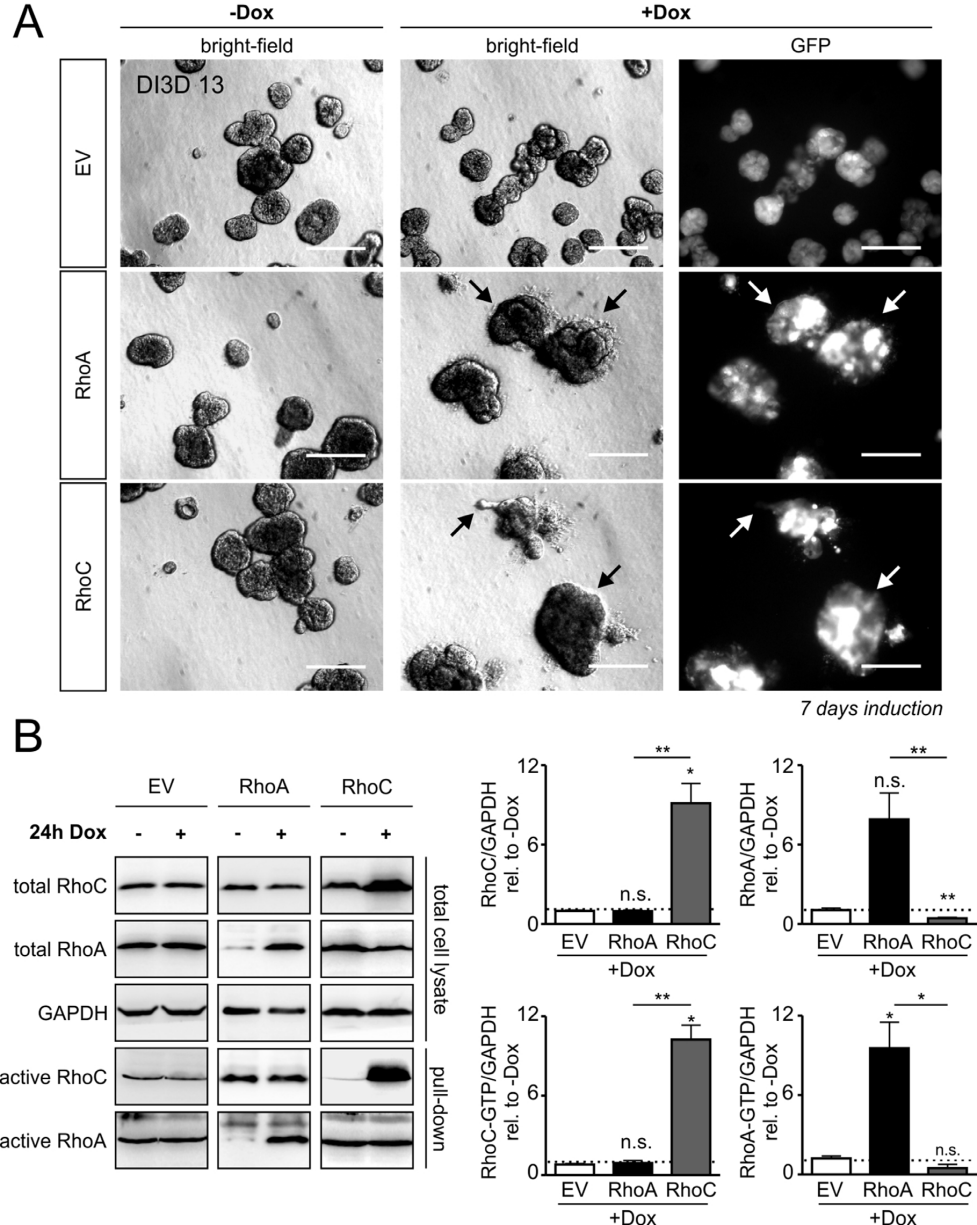
Doxycycline-inducible overexpression of RhoA and RhoC in MCF-10A cells (**A**) MCF-10A cells were transfected with solely GFP (EV, top), RhoA plus GFP (RhoA, middle) or RhoC plus GFP (RhoC, bottom) -containing constructs under the control of the tet-ON promoter. The transgenic MCF-10A cell lines were left untreated or were stimulated for GFP/Rho expression by addition of Doxycycline (+Dox), DI3D 7-13. GFP-expression is shown in all three induced transgenic cell lines (right). There was no GFP expression detectable in non-induced cells; scale bar 50 μm. (**B**) Typical Western-blots determining the expression levels of total and active RhoA and RhoC proteins in GFP-, RhoA- and RhoC-expressing MCF-10A cells (left). Quantification of total RhoC (top left), active RhoC-GTP (bottom left), total RhoA (top right) and active RhoA-GTP (bottom right) after 24 h of gene induction. Protein levels were normalized to the reference protein GAPDH and are given relative to non-induced control cells. The shown western blot results of RhoA, RhoC and EV induction are derived from different gels as indicated by the black framing. Note that RhoC overexpression significantly suppresses total RhoA levels. Bars and error bars represent mean (*N* = 3) ± SEM. ^*^p < 0.05; ^**^p < 0.01; ^***^p < 0.001; n.s., *p* ≥ 0.05; two-tailed, one-sample *t*-test.

We seeded MCF-10Atet cells with the empty vector (only GFP-encoding, EV), the RhoA- and the RhoC-encoding vector, respectively, into Matrigel/Collagen I and incubated the cells for 6 days until acini were formed. Then, we induced the expression of Rho proteins by addition of doxycycline. Successful induction of gene synthesis was controlled by microscopy of the co-expressed GFP. In contrast to toxin treatment, neither induction of RhoA expression nor expression of RhoC was sufficient to induce invasion. However, RhoC- and to a smaller extent RhoA overexpression induced a morphologically premalignant phenotype characterized by irregular borders, clustered structures and individual escaping and blebbing cells (Figure 3A, bright-field micrographs on the right, arrows). Expression of Rho proteins was validated by Western-blotting (Figure 3B).

To check why the acini appeared larger, we analyzed the influence of Rho overexpression and CNF intoxication on the proliferation in all cell lines (Supplementary Figure 3). Interestingly, we could demonstrate a decreased ability in colony formation of Rho-overexpressing as well as of CNF-intoxicated cell lines (Supplementary Figure 3A). This was further confirmed by analysis of the doubling time, which was increased through all toxin treatments as well as by Rho overexpression (Supplementary Figure 3B). As occurrence of cell death and senescence may influence these results, we investigated these processes by TUNEL and β-Galactosidase stainings, respectively. Here, we found that RhoA as well as RhoC overexpression induced a slightly increased induction of cell death, which might further explain the blebbing phenomenon. However, increased cell death could not be demonstrated in case of the intoxicated cell lines (Supplementary Figure 3C). Similarly to results of Vannini et al., we found an underlying increased level of senescence through CNF toxin treatment as shown by cytochemicalstainings in monolayer and 3D cultures [31] (Supplementary Figure 3D).

### Knockdown of RhoC but not knockdown of RhoA reduced toxin-induced invasion

**Figure 4:**
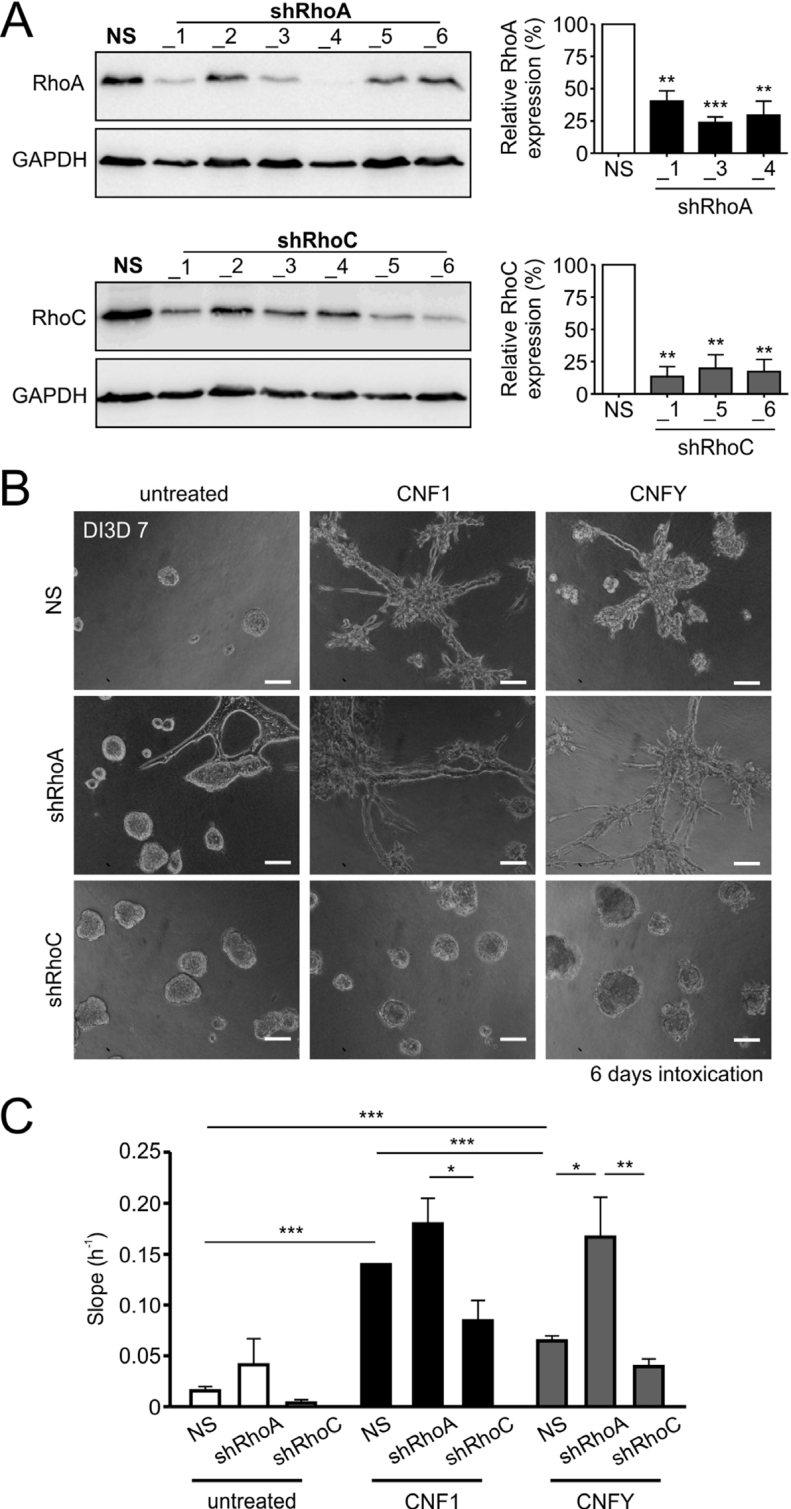
Knockdown of RhoC but not RhoA precludes the pro-invasive effect of CNF1- and CNFY treatment (**A**) Left: Western-blots showing the knockdown efficiency for six constitutively expressed shRNA constructs against RhoA (shRhoA, top) and RhoC (shRhoC, bottom) plus a non-silencing control construct (NS) in WT-MCF-10A cells. Right, corresponding statistical analysis of the three most effective constructs (*N* = 4). Although not the most effective, shRhoA_3 was chosen for knockdown of RhoA in further experiments, because it was already used before [58]. (**B**) Phase-contrast micrographs of MCF-10A 3D cultures (DI3D7) transfected with the non-silencing (NS, top), shRhoA (middle) or shRhoC constructs (bottom). Cultures were grown in untreated control conditions (left) or treated with 1 nM CNF1 (middle) or CNFY (right) during DI3D1-7. Transfection with shRhoC but not shRhoA antagonizes the pro-invasive effect of CNF1/Y treatment. Scale bar 50 μm. (**C**) Quantification of the functional invasiveness by the Xcelligence^®^ device. MCF-10A cells transfected with NS, shRhoA or shRhoC were analyzed under untreated control conditions (white bars) or treated with either 1 nM CNF1 (black bars) or 1 nM CNFY (grey bars). Bars and error bars represent mean ± SEM. ^*^p < 0.05; ^**^p < 0.01; ^***^p < 0.001; two-tailed, one-sample *t-test* and one-way ANOVA.

To analyze whether one of the GTPases would be necessary for invasion, we decided to use a shRNA-mediated knockdown. As shown in Figure 4A, each of the six used shRNAs efficiently knocked down either RhoC or RhoA, respectively. From analyses of four independent experiments (Figure 4A right), we chose shRNA_3 for knockdown of RhoA and shRNA_6 for knockdown of RhoC. In the subsequent 3D invasion experiments, loss of RhoC but not reduction of RhoA expression levels completely inhibited toxin-induced invasion (Figure 4B). In line with this, knockdown of RhoC significantly reduced the invasive potential of MCF-10A cells in the impedance measurements as well (Figure 4C). Interestingly, knockdown of RhoA even increased toxin-induced invasion. This may be due to the fact that knockdown of RhoA increased the level of RhoC in MCF-10A cells in our experiments (Supplementary Figure 4). The data indicate that RhoC is required for toxin-induced invasion, whereas RhoA seems to have no impact on it.

### RhoA and RhoC differently influence the gene expression profile

To study the basis for RhoC dependence of invasion, we performed a comparative analysis of the transcriptome response of MCF-10A cells expressing either GTPase or treated with the toxins using Illumina Human v4 bead arrays. To this end, cells were stimulated with doxycycline or treated with the toxins for one and seven days and lysed thereafter.

**Figure 5:**
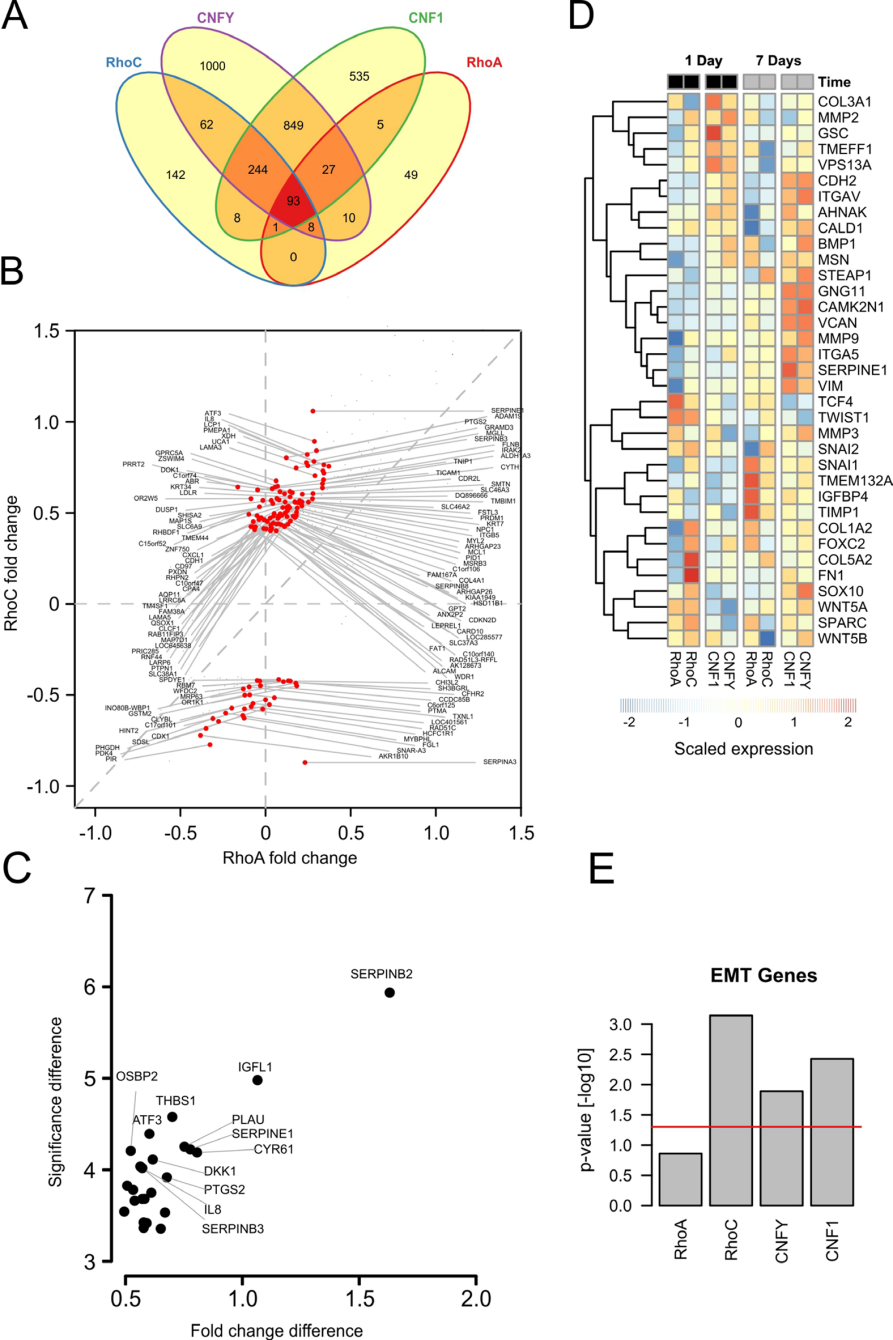
Transcriptome analysis shows induction of EMT genes specifically for RhoC overexpression (**A**) Venn diagram of differentially regulated genes after 24 hours of RhoC or RhoA expression, and CNF1 or CNFY treatment (adjusted *p-value* < 0.05). (**B**) Scatterplot depicting all genes from (A) that were differentially regulated under overexpression or RhoA or RhoC. Genes having a fold change > 0.3 (log2) are additionally marked in red and denoted by their gene symbol. (**C**) One-sided volcano plot showing the significantly up-regulated genes in RhoC compared to RhoA overexpressing cells after one day versus the difference of the (−log_10_) transformed *p-value* for differential regulation. All genes having a significance difference > 4 are annotated with their gene symbol. (**D**) Heatmap showing the scaled expression of EMT related genes after one and seven days of RhoA/RhoC overexpression or CNF1/CNFY treatment. Genes were hierarchically clustered by their Euclidean distance using complete linkage. (**E**) Gene set enrichment of EMT activating genes after one day of RhoA/RhoC overexpression or CNF1/CNFY treatment. The bars indicate the (−log_10_) transformed *p-value*.

As shown in the Venn diagram in Figure 5A, both CNFY and CNF1 shared the majority of their significantly upregulated genes (adjusted *p-value* < 0.05 and log2 fold change > 0.2) with RhoC- but not with RhoA-overexpressing cells (337 vs. 120 genes). To investigate whether this gene overlap is associated with cell invasion, we first checked the presence of epithelial-mesenchymal transition (EMT), which is often associated with tumor cell migration and invasion. A gene set enrichment on EMT genes showed a significant up-regulation for the toxins and RhoC after one day already, but not for RhoA overexpression (Figure 5D and 5E). RhoA overexpression induced a significant upregulation of some EMT genes (for example: tissue inhibitor of metalloproteases 1 (TIMP1)) after seven days, albeit the expression profile differed from that of RhoC (see Figure 5D, columns 5 and 6). To obtain a detailed insight, we plotted all genes that were significantly regulated after one day in either RhoC or RhoA (Figure 5B, 5C, compare also Supplementary Table 2). Among the transcripts that were consistently upregulated after RhoC but not RhoA induction, we found PLAU (Plasminogen activator, urokinase), SERPINE1 (Serpin Peptidase Inhibitor, Clade E, Member 1) as well as PTGS2 (Prostaglandin-Endoperoxide Synthase 2, Cyclooxygenase 2, COX-2), for example. Interestingly, these genes have been previously linked to migration of primary human keratinocytes as well as to differentiation of PC12 cells [32, 33]. The induction of pro-migratory genes of interest by toxin treatment was additionally validated by qRT-PCR (Supplementary Figure 6). The identified genes were reproducibly upregulated by Rho stimulation. For more information of all regulated genes, the microarray data, including the raw data, had been uploaded to Gene Expression Omnibus with the GEO ID GSE98244. Moreover, regulated genes are shown in Supplementary Table 2.

### Inhibition of COX-2 is sufficient to reduce toxin-induced invasion

**Figure 6:**
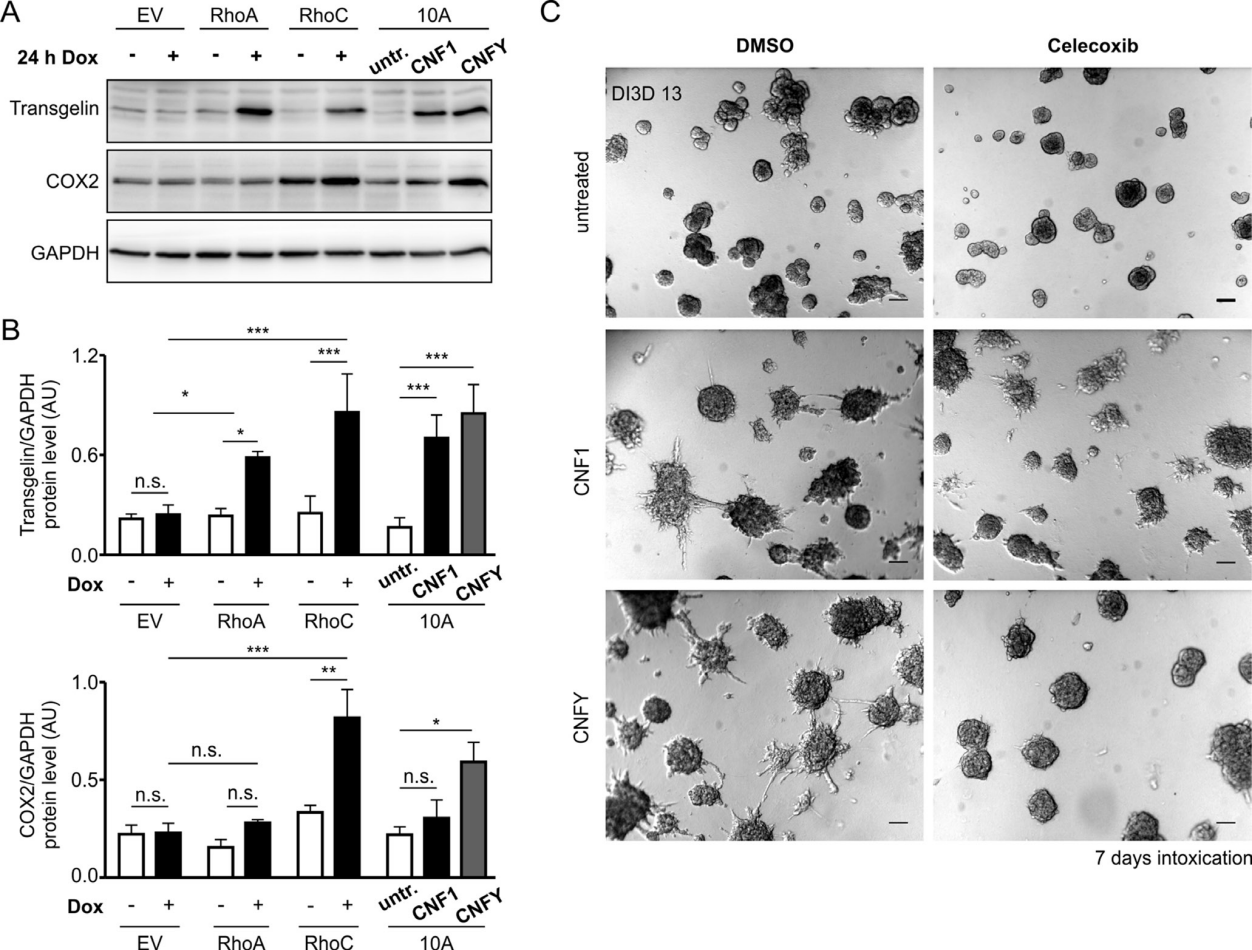
Transgelin and COX-2 as target genes of Rho proteins and CNF toxins (**A**) Western-blots showing the expression levels of Transgelin and COX-2 as putative candidate target genes of RhoA/C and CNF1/Y. Protein levels were determined in GFP-, RhoA- and RhoC-inducible MCF-10A cells ± Doxycycline induction over 24 h (left) and in WT-MCF-10A cells with or without treatment with 1 nM CNF1 or CNFY over 24 h (right). (**B**) Average protein expression levels (*N* = 3) in the different cell lines and treatments as described in (A) for Transgelin (top) and COX-2 (bottom) normalized to GAPDH. (**C**) Representative phase-contrast micrographs of untreated (top), CNF1- (middle) and CNFY-treated (bottom) MCF-10A 3D cultures, which were additionally treated with the specific pharmacological COX-2 inhibitor (20 μM Celecoxib, right) or DMSO (left) as a control (pictures taken at DI3D 13, CNF1/Y treatment DI3D 7-13, DMSO/Celecoxib treatment DI3D 7-13), scale bar 50 μm. Bars and error bars represent mean ± SEM. ^*^p < 0.05; ^**^p < 0.01; ^***^p < 0.001; n.s., *p* ≥ 0.05.

Most of the genes and proteins were induced by both RhoC and RhoA expression (Supplementary Table 2). Because we found Transgelin to be by far the strongest differentially regulated gene following expression of RhoC (compare Supplementary Table 2), we included a Western blot analysis to demonstrate its induction on the protein level by both GTPases, as well as following treatment with CNF1 or CNFY (Figure 6A, 6B). In fact, only one gene turned out to be preferentially and robustly upregulated by RhoC induction (0.8 log2 fold after 1 day of stimulation, Supplementary Table 1) but less by RhoA overexpression (0,1 log2 fold induction after 1 day, Supplementary Table 1): PTGS2 (COX-2), that is known to increase cell motility and invasion in breast cancer cells [34]. To confirm the involvement of COX-2 in toxin-induced invasion of MCF-10A cells, we treated preformed MCF-10A acini with the toxins in the presence and absence of the specific COX-2 inhibitor Celecoxib. As shown in Figure 6C, the invasive phenotype induced by the toxins was significantly reduced in the presence of the COX-2 inhibitor.

## DISCUSSION

Rho GTPases are involved in central aspects of tumorigenesis, including the regulation of the actin and tubulin cytoskeleton as well as gene regulation and survival. Therefore, it is not surprising that deregulation of these molecular switches has a major impact on tumor formation and metastasis, which includes several cellular mechanisms like proliferation, dissociation of cell-cell contacts and proliferation. Rho GTPases are main regulators of these processes. Several mutations of Rho GTPases have been found in tumor tissues. However, compared to Ras, Rho mutations are rare (http://www.cbioportal.org). Moreover, some of the identified mutations are rather functionally inactivating. This suggests that Rho GTPases are no general drivers of cancer development, but still they might support tumor formation in a more specific manner. In metastatic breast cancer, RhoA deletions are more frequent than amplifications (5% versus 1%), whereas RhoC is exclusively upregulated (2.3%).

To analyze the role of RhoA and RhoC, we first used two bacterial toxins to activate the endogenous pool of Rho GTPases in benign, non-invasive breast epithelial cells (MCF-10A). However, one has to keep in mind that CNF-modified Rho proteins may be ubiquitinated and degraded by the proteasome [35–37]. Second, we combined toxin treatment with specific knockdown of RhoA and RhoC, respectively. Third, we generated isogenic stable cell lines, in which doxycycline treatment induces the expression of RhoA or RhoC. We chose an inducible system to minimize effects of permanent overexpression in the best possible manner. Overexpression may influence the sensitive balance between the levels of Rho GTPases and GDIs [38, 39]. Moreover, it may change the set of effector molecules activated, because their binding to Rho GTPases is based on slight differences in affinity [38]. An inducible system, however, allows the increased expression after acini have been formed, which more closely resembles the *in vivo* situation. Indeed, we could show that activation of Rho GTPases is sufficient to induce an invasive phenotype of acini formed by benign breast epithelial cells.

However, expression of neither RhoA nor RhoC overexpression alone had a major effect on morphological invasiveness as assessed in 3D cultures.

Remarkably, it is well established that the expression of oncogenes in benign epithelial cells may come along with the induction of wildtype p53 and, accordingly, activation of a senescence program through the induction of p21. This fits to our results: CNF toxins induce a broad invasive gene signature in contrast to RhoC induction alone, through which tumor-control mechanisms become active as well. The induced cell death in RhoA and RhoC overexpressing cells could be linked to the hyper-activation of ROCK. Inhibition of ROCK through treatment with the selective ROCK inhibitor H1152 in parallel to Rho overexpression normalized the increased Caspase 3/7 activation to basal levels again (Supplementary Figure 5).

In former studies we showed that treatment of epithelia with CNF1 reduced the epithelial barrier function [40]. Therefore, dissociation of cell-cell contacts may be relevant for toxin-induced release of single cells from the preformed acini. Interestingly, only knockdown of RhoC was able to repress the toxin-induced disruption of the epithelial integrity and to inhibit invasion.

Knockdown of RhoA had no effect. This indicates that RhoC is more relevant for induction of invasion. In line with this, depletion of RhoC reduced metastasis in mice [41]. The comparative analysis of the gene expression profiles and its validation by Western-blot and qPCR revealed only one gene predominantly upregulated by RhoC: PTGS-2 (COX-2), the inducible form of cyclooxygenase (COX). Overexpression of COX-2 promotes invasion, whereas depletion of COX-2 reduced neoplastic growth in APCdelta716 mice, a model for colorectal cancer [42]. COX-2 inhibitors are also suggested to be beneficial in other cancer entities like lung cancer [43, 44], gastric cancer [45] or acute leukemia [46]. There is considerable evidence that prostaglandins play a role in cancer development [47].

COX-2 induces the synthesis of prostaglandin E-2. Selective inhibition of PGE-2 receptors inhibits invasion of endometrial epithelial cells by suppression of the synthesis of matrix metalloproteases [48]. This suggests an autocrine loop of RhoC, COX-2, PGE-2, PGE2R, MMPs since in our model no other cells are present. Indeed, MMP3 and MMP9 expression is upregulated by stimulation of Rho GTPases in MCF-10A cells (compare Figure 5). Consistently, specific inhibition of COX-2 by Celecoxib in our experiments significantly reduced the invasive phenotype of MCF-10A cells by the Rho activating toxins. However, more studies analyzing specific and unspecific COX-inhibitors are required to study the impact of cyclooxygenases on invasion and metastasis. Indeed, there has been increasing interest in the use of selective COX-2 inhibitors for prevention or treatment of breast cancer with promising results in preclinical studies [49]. However, epidemiological studies with users of non-specific COX-inhibitors like aspirin [50] showed only a slight reduction of the breast cancer incidence rate. Studies analyzing add-on therapy with low numbers of patients showed no additional treatment effect [51]. So far, evidence for a protective or therapeutic effect of COX-2 inhibitors is still limited. However, since RhoC is predominantly upregulated in inflammatory breast cancer [52], COX-2 inhibitors may be especially useful in the treatment of specific breast cancer patients. Therefore, clinical studies using COX-2 inhibitors with disease-specific subgroup analysis and patient selection are urgently required.

## MATERIALS AND METHODS

### Cells and transfection methods, inhibitors, inducers and CNF toxins

The mammary epithelial MCF-10A wild-type cell line was purchased from ATCC. MCF-10Atet cells, constitutively expressing a second generation Tet-regulated transcriptional transactivator and silencer, have been described in detail previously [29]. Both wild-type and stably transfected cell lines were maintained in growth medium consisting of DMEM/F12 supplemented with 5% horse serum, 10 μg/ml insulin, 0.5 μg/ml hydrocortisone, 20 ng/ml EGF and 0.1 μg/ml cholera toxin. Stable cell lines inducible for Rho overexpression were generated via nucleofection (AMAXA^®^) with the *AhdI*-linearized pTET-bsr vector containing wild-type cDNA of RhoA or RhoC along with GFP and promoter binding sites for the Tet-regulated transcriptional transactivator (for further details refer to [29, 53]). Knockdown of Rho GTPases was achieved through constitutive expression of shRNAs against RhoA and RhoC (lentiviral pGIPZ vectors were obtained from GE-Dharmacon). Doxycycline for the induction of transgenic overexpression was purchased from Sigma and used at 2 μg/ml. Inhibitors against ROCKI/II, myosin II and COX-2 were ordered from Enzo or Sigma and dissolved in DMSO. CNF toxins were purified as described previously and used at 1 nM for the indicated time periods [26, 54]. Inhibitors, toxins and inducers were renewed every 2-3 days with every medium exchange.

### Rho activity assay and Western-blot analysis

Rho activity was determined via Rho effector pulldown assay and subsequent Western-blot analysis. Briefly, beads-coupled GST-Rhotekin-RBD or GST-PAK-RBD were incubated for 1 h with the respective cell lysate. An aliquot of the total cell lysate served as input control. After several washing steps, beads-bound Rho was separated by Sodium dodecylsulfate polyacrylamide gel electrophoresis (SDS-PAGE) and analyzed by Western-blotting. Blot membranes were blocked in 5% milk powder or 5% BSA in TBS-T and incubated at 4°C overnight with one of the following antibodies: RhoA (67B9) rabbit, RhoC (D40E) rabbit and a suitable secondary antibody coupled to horseradish peroxidase (HRP).

Further antibodies used for Western-blotting: COX-2 (D5H5) XP^®^ rabbit monoclonal antibody (mAb; NEB), GAPDH (6C5) mouse mAb (EMD-Millipore) and Transgelin/SM22 (2A10C2) mouse mAb (Proteintech).

### 3D cultures

Overlay 3D cultures were set up as reported previously with slight modifications [30]. Briefly, cells were trypsinized, suspended in 2% Matrigel (growth factor-reduced basement membrane; Corning)/Assay medium mixture and seeded as single cells on top of preformed, polymerized Matrigel/collagen I beds. In case of the matrix degradation assay, DQ collagen I was added at 25 μg/ml to the Matrigel/collagen mixture immediately before coating (Life Technologies). For long-term cultivation of 3D cultures, medium supplemented with 2% Matrigel was exchanged every 2-3 days.

For immunofluorescence, 3D cultures were fixed in 4% PFA for 15 min and stained according to Debnath et al. [30]. DAPI was used as a counterstain for nuclei. For fluorescence staining, the primary antibody anti-Laminin-V γ2 chain (D4B5) mouse mAb from EMD-Millipore and actin-labelling Rhodamine-Phalloidin from Hypermol were used. TUNEL-labeling of dead or β-Galactosidase-expressing senescent cells in 3D or monolayer cultures was performed at the indicated time points according to the manufacturer's instruction (*In Situ* Cell Death Detection Kit, TMR Red, Roche; Senescent Cells, Histochemical Staining Kit, Sigma).

Confocal microscopy of stained cells was performed on a Zeiss LSM 510 equipped with a Plan-Apochromat 63x (N.A. 1.2, oil) and a Plan Apochromat 20x (N.A. 0.75). For image analysis ZEN 2010 (Zeiss) software and Image J were used.

### Cell-based assays

Induction of caspase activity was determined in monolayer cultures according to the manufacturer's instruction (Apo-ONE^®^ Homogeneous Caspase-3/7 Assay). For colony formation, 500 cells were homogenously seeded into 6-well plates. After 8-12 hours of attachment, doxycycline or CNF toxins were added for intoxication or overexpression of Rho GTPases and renewed every 2-3 days with medium exchange. At day 8 of cultivation, grown colonies were fixed and stained for 30 min with crystal violet (0.5% wt/vol in dH_2_O). After washing and drying, stained area intensities from scanned well plates were determined with the help of the *colony area* plugin by ImageJ.

### Cell proliferation assay

Cell doubling time was calculated from a 2-time point calculation over 48 hours according the following formula:

Doubling Time = Duration ^*^ ln(2)/ln(final concentration)−ln(inital concentration)

In brief, cells were seeded at 50,000 per well and allowed to attach and reach a stable proliferation rate for 18 h initially. Then, for a subset of cultures, cell concentration was determined for the first time and remaining duplicate cultures were treated as indicated with doxycycline or toxins. After a proliferation time of further 30 h, cell concentrations for the second time point were determined.

### Invasion assay

Cell invasion through Matrigel was monitored by real-time impedance measurement via the xCELLigence^®^ system using Cell Invasion and Migration (CIM) plates. In case of invasion assessment, the upper side of the membrane was coated with a 1:40 diluted Matrigel/collagen I mixture in serum-free medium that was allowed to polymerize for 4 h at 37°C. After addition of serum-containing medium to the lower and serum-free medium to the upper chamber, a serum gradient is established along the membrane. Cells were seeded as a single cell suspension at 1 × 10^5^ cells per well into the upper chamber and after a short time of settlement monitored for invasion. Resulting impedance values were recorded by electrodes over the time and analyzed for their slopes in the linear range, which are directly proportional to the rate of invading cells.

### RNA isolation and qRT PCR

RNA was isolated from 3D cultures at indicated time points via the RNeasy Mini Kit from Qiagen according to the manufacturers’ instruction. In brief, medium was removed, cells were lysed and Matrigel was homogenized. Cell lysates were diluted 1:1 in 70% ethanol and added to the RNeasy columns. After centrifugation at 8,000 g for 30 s, columns were washed three times, centrifuged another time and RNA was eluted in 35 μl RNAse-free dH_2_O. Final RNA concentration was determined photometrically at 260 nm. Samples of 1 μg RNA were used for further transcriptomic analysis or subjected to qRT-PCR. As a first step, cDNA synthesis was carried out using the QuantiTect Reverse Transcription Kit from Qiagen according the manufacturers’ instruction. Afterwards, cDNA was diluted at 1:10 and amplified using the GoTaq^®^ qPCR Master-Mix according to protocol (Promega) on a Mastercycler^®^ Realplex (Eppendorf). Raw data was analyzed with LinRegPCR 2012. GAPDH served as a reference gene.

### Transcriptomic profiling or Microarray analysis

Total RNA was extracted in biological duplicates from (i) CNF1- and CNFY-treated and untreated MCF-10A organoids at one and seven days after intoxication and from (ii) organoids transfected with an inducible RhoA- or RhoC-encoding vector with or without addition of doxycycline at days one and seven after induction. Biotinylated cRNA were prepared with the Ambion MessageAmp kit for Illumina arrays according to the manufacturer's protocol. Quality of cRNA was controlled using RNA Nano Chip Assay on an Agilent 2100 and hybridized to Illumina HumanHT12-v4 BeadChips (Illumina, San Diego, CA, USA) according to the manufacturer's protocol. Raw microarray data were chip-wise processed using the Bioconductor R package beadarray [55] and subsequently quantile normalized together. Illumina Probes were mapped to reannotated Entrez IDs using the Illumina Human v4 annotation data (Version 1.26) from Bioconductor. If several probes mapped to the same Entrez ID, the one having the largest interquartile range was retained. Microarray data have been deposited in GEO under the access ID GSE98244. Differential gene expression analysis between treatment groups was calculated using the R limma package [56]. Gene set enrichment analyses were performed with GAGE algorithm [57], which tests whether a gene set is highly ranked relative to other genes. For functional annotation, we used genes sets from the human gene ontology (GO) as provided by the R/Bioconductor org.Hs.eg, db package (Version 3.1.2). Only gene sets having more than 5 and less than 500 genes were considered to retain those that are statistically robust and biologically informative.

### Tissue specimens and immunohistochemistry

The study included formalin-fixed and paraffin-embedded (FFPE) tissue specimens from 9 patients. Matched normal breast epithelia (*n* = 9), ductal carcinoma *in situ* (DCIS; *n* = 9) and ductal breast carcinomas (*n* = 9) were analyzed. The study was approved by the local institutional ethic committee (#324; Ethik-Kommission der Albert-Ludwigs-Universität, Freiburg, Germany).

For immunohistochemistry, the FFPE tissue specimens were cut at 3 μm thickness, deparaffinized and subjected to antigen retrieval in pH 6.1 citrate-buffer. Subsequent staining was done by incubation with a primary RhoC antibody (1:20000; rabbit monoclonal, clone D40E45; Cell Signalling Technologies) or RhoA antibody (1:20000; rabbit monoclonal, clone 67B9; Cell Signalling Technologies), respectively and a secondary antibody and LSAB-Fast-Red detection system according to standardized procedures using a DAKO Autostainer (all DAKO Cytomation, Glostrup, Denmark). RhoC protein expression was evaluated as negative or positive by co-evaluation of sub-cellular localization.

### Statistical analysis

Statistical analysis was performed with Prism 5.0. Student's or One-Sample *t*-test was used for two-sample and ANOVA for group comparisons. Tests were performed two-tailed if not stated otherwise. Values given in the text, bars and error bars in the figures are representing means ± standard error of the mean (SEM).

## SUPPLEMENTARY MATERIALS FIGURES AND TABLES




