# Supplementary material for: Specific role of RhoC in tumor invasion and metastasis
Source: Oncotarget. 2017 Sep 16;8(50):87364–78. doi: 10.18632/oncotarget.20957 (PMC5675639; doi:10.18632/oncotarget.20957)
Supplement: Supplementary file 1 [file oncotarget-08-87364-s001.pdf]

Specific role of RhoC in tumor invasion and metastasis

SUPPLEMENTARY MATERIALS

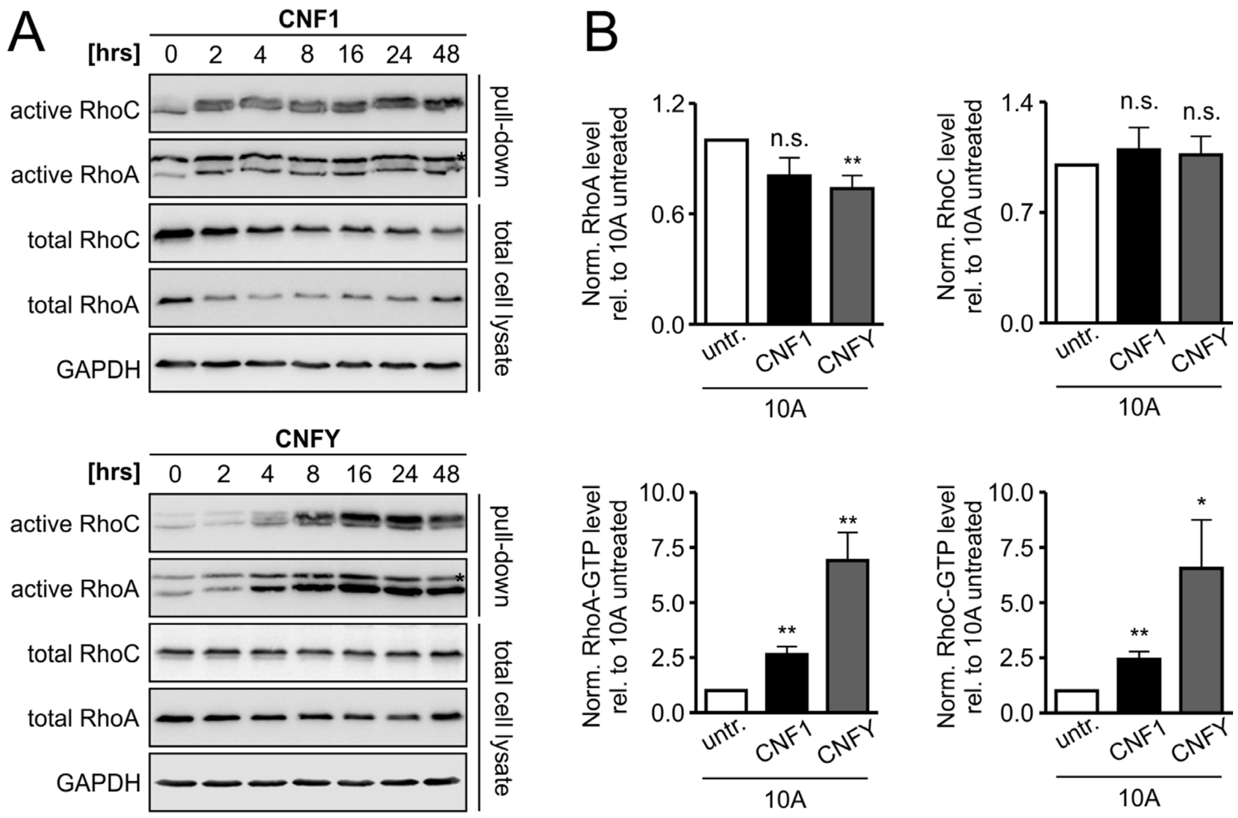

Supplementary Figure 1: Time course of Rho protein activation by toxin treatment. (**A**) Western-blots showing the expression levels of the total amount and only the active, GTP-bound form of RhoA and RhoC over the course of 48 h treatment with CNF1 (top) or CNFY (bottom) in WT-MCF-10A cells. Asterisks indicate unspecific labeling by the anti-RhoC and anti-RhoA antibodies. (**B**) Average protein expression levels (N ≥ 6) of total (top) and active (bottom) RhoC (left) and RhoA (right) in control cells, CNF1- and CNFY-treated WT-MCF-10A cells 24 h after start of toxin treatment. Protein levels were normalized to the reference protein GAPDH and are given relative to the untreated control cells. Bars and error bars represent mean ± SEM. *$p < 0.05$; **$p < 0.01$; n.s., $p \geq 0.05$; two-tailed, one-sample *t*-test.

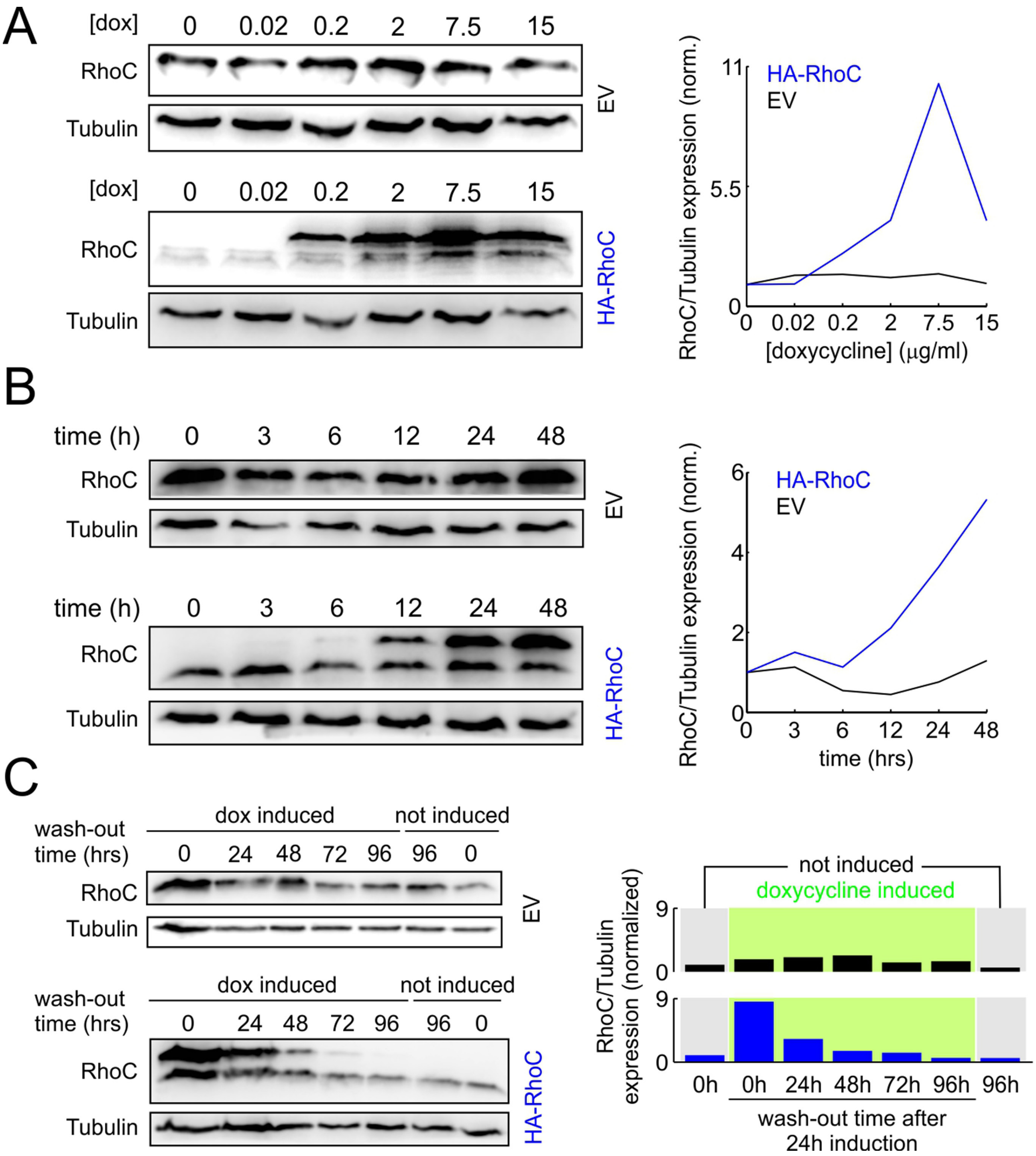

Supplementary Figure 2: Characterization of the Doxycycline-inducible system. MCF-10Atet cells were transfected with two different vector constructs encoding either GFP alone (black) or both, GFP and RhoC (blue; HA-tagged RhoC). (**A**) Doxycycline concentration-dependent induction is shown by quantitative Western-blot analysis for RhoC and tubulin after stimulation with the indicated doxycycline concentrations for 24 h. (**B**) Time-dependent induction was assessed through stimulation of transfected cell lines with 2 μg/ml doxycycline for the indicated time periods and analyzed by quantification of RhoC levels relative to tubulin expression. (**C**) Switching off the inducible system is demonstrated by doxycycline wash-out. MCF10A-tet cells were induced with 2 μg/ml doxycycline for 24 h. After wash-out of doxycycline, total cell lysates were prepared after indicated time periods and subjected to Western-blot analysis for RhoC and tubulin. Note that RhoC expression levels decayed back to the level of non-induced cells after 72 h following wash-out as shown in the right quantification. Left, representative Western-blot results (A-C).

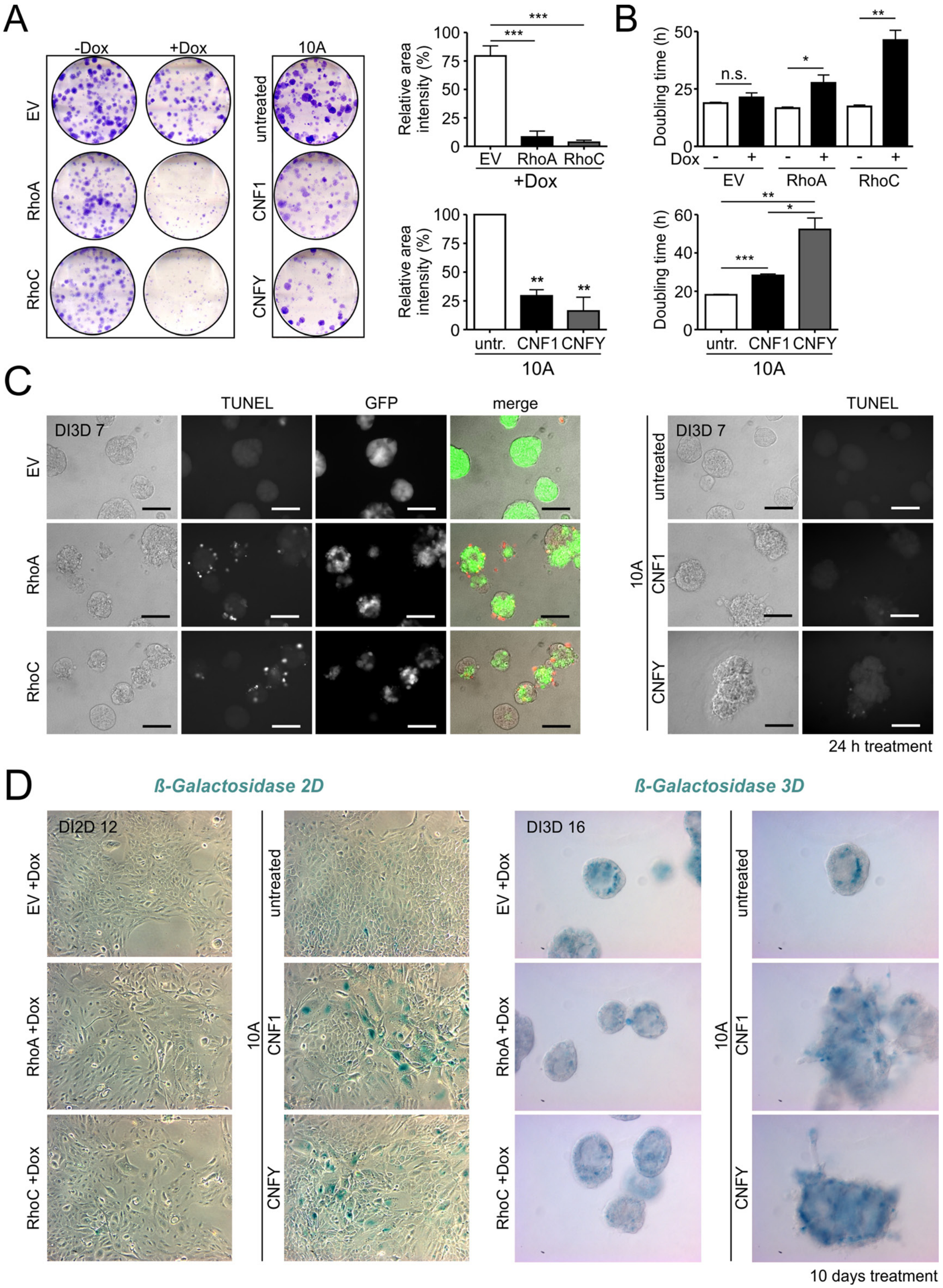

Supplementary Figure 3: Proliferation, cell death and senescence in RhoA/C-overexpressing and CNF1/Y-treated MCF-10A cells. (**A**) Crystal violet-stained colonies of GFP-, RhoA- and RhoC-expressing MCF-10A cells at DI2D 8 with or without induction by 2 µM Doxycycline (±Dox, DI2D 2-8) (left). Middle, untreated or 1 nM CNF1/Y-treated (DI2D 2-8) WT-MCF-10A cells at DI2D 8. Right, quantification of the Crystal violet-stained area as a measure of colony formation. Top, colony-formation assay with RhoA- and RhoC-overexpressing cells relative to their non-induced counterparts ($N = 6$). Bottom, treatment of WT-MCF-10A cells with CNF1 and CNFY ($N = 3$). (**B**) Analysis of the cell doubling time of RhoA- and RhoC-overexpressing MCF-10A cells (Dox induction for 30h, top) or after CNF1 and CNFY treatment (30 h, bottom), both relative to the non-induced control cells ($N = 3$ experiments). (**C**) Left, TUNEL staining of RhoA- and RhoC-expressing or GFP-expressing MCF-10A 3D cultures (DI3D 7, Dox 24 h). Right, analogous analysis for untreated (top), CNF1- (middle) and CNFY-treated (bottom) WT-MCF-10A cells (DI3D 7, toxin treatment 24 h). (**D**) Staining of acidic β-Galactosidase in 2D- (top, DI2D 12) and 3D cultures (bottom, DI3D 15) of Doxycycline-induced GFP- (EV+), RhoA- (RhoA+) and RhoC-expressing MCF-10A (RhoC+) cells, and untreated (10A), CNF1- (10A-CNF1) and CNFY-treated (10A-CNFY) WT-MCF-10A cells (treatment with Dox or CNF1/Y: 10 days) $^{*}p < 0.05$; $^{**}p < 0.01$; $^{***}p < 0.001$; two-tailed, one or two-sample t-test.

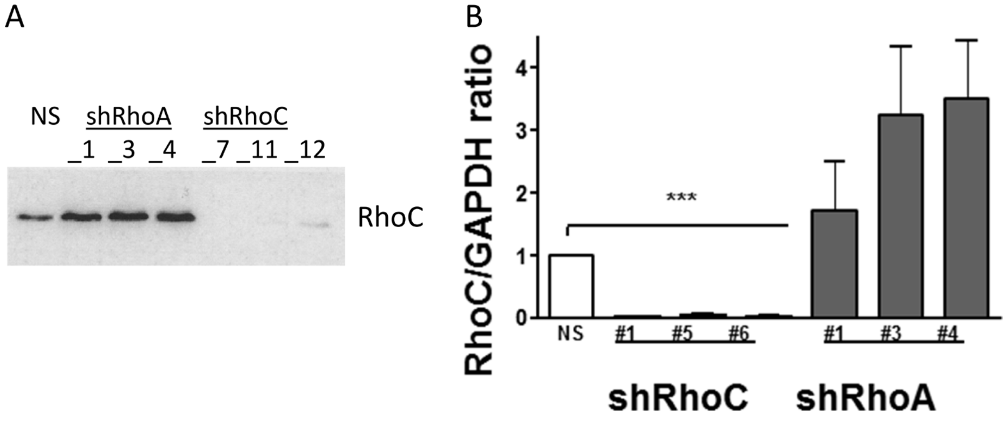

Supplementary Figure 4: Knockdown of RhoA increases the level of RhoC.

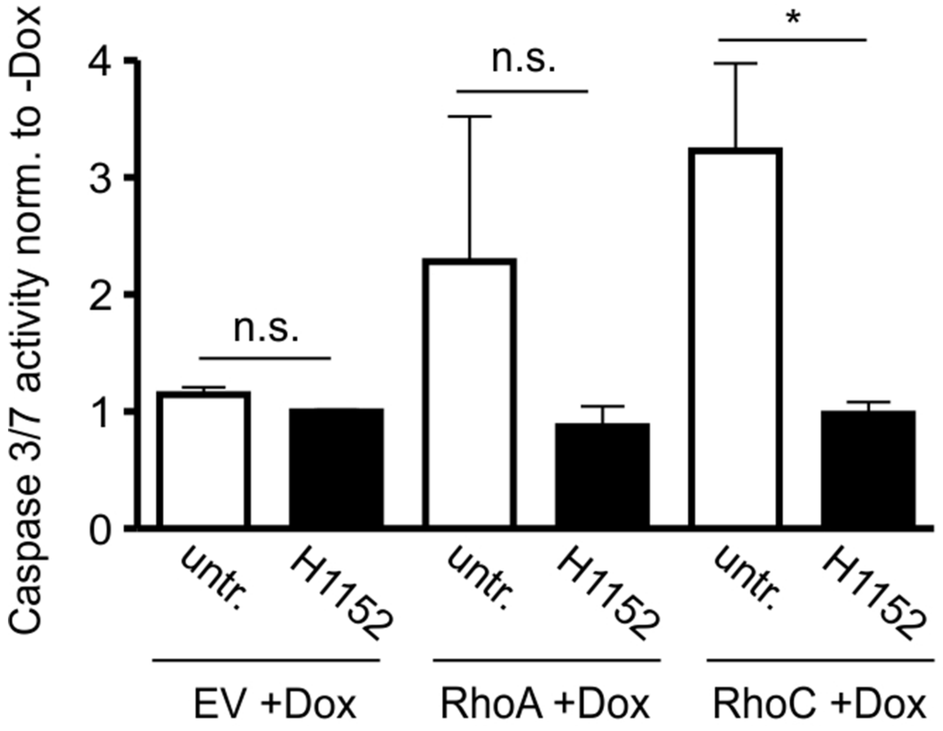

Supplementary Figure 5: Apoptosis induced by Rho protein overexpression is mediated by the Rho kinase (ROCK). Caspase 3/7 activity as a measure for apoptosis induction was determined in GFP-, RhoA- and RhoC-expressing MCF-10A cells with or without treatment with the ROCK inhibitor H1152 (H1152 treatment 20 h, Dox induction 24 h). RhoA and RhoC overexpression lead to higher Caspase 3/7 activity, which can be prevented by treatment with H1152. Bars and error bars indicate mean ± SEM of $N = 3$ separate experiments. *$p < 0.05$; one-tailed, two-sample *t*-test.

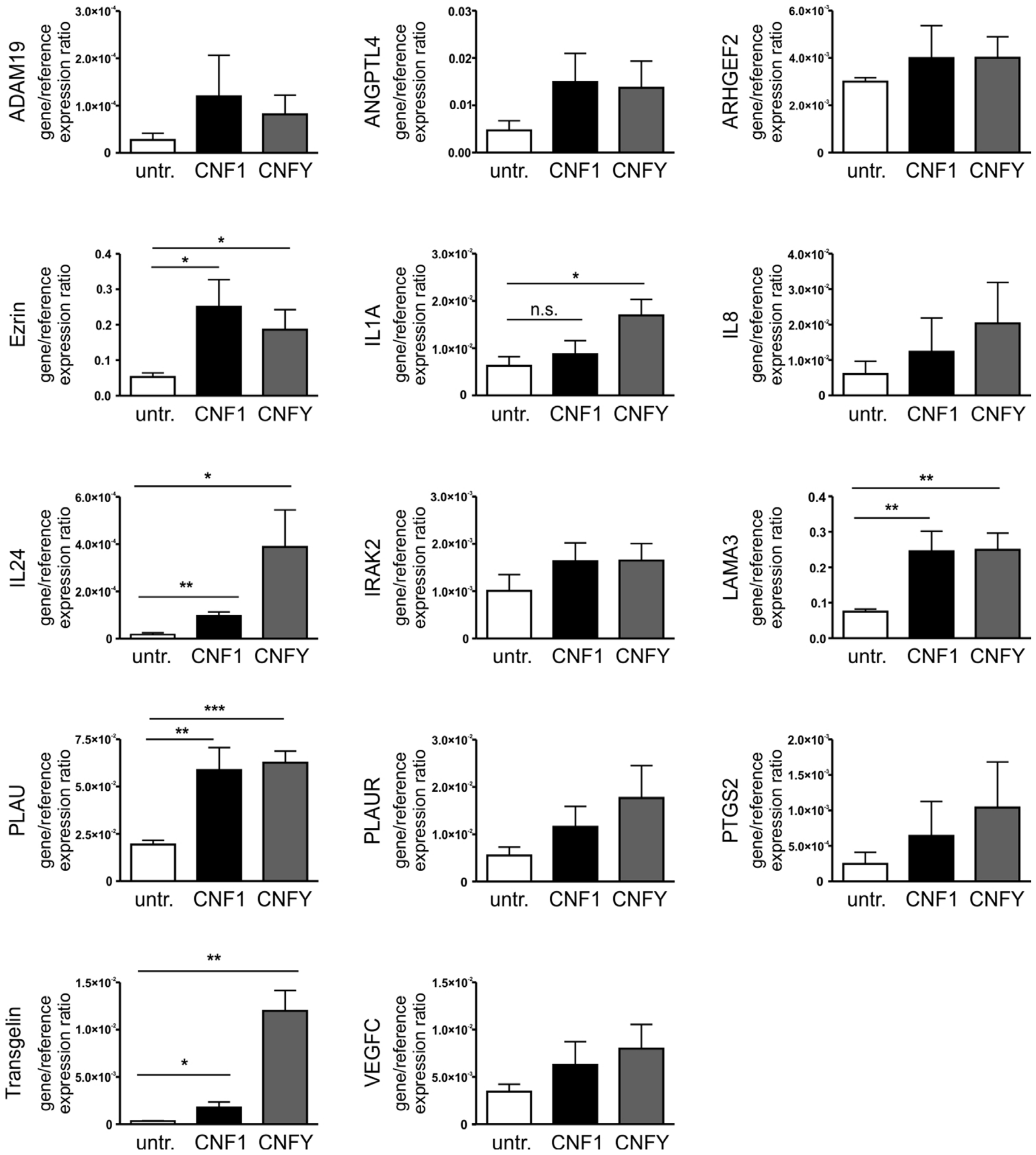

Supplementary Figure 6: Regulation of differential gene expression through Rho activation. Transcriptomic data were validated by qRT-PCR of various differentially expressed target genes in MCF10A 3D acini that were treated for 24 h with 1 nM CNF1 or CNFY and compared to corresponding control cells. Bars and error bars indicate mean ± SEM of N ≥ 3 experiments. *$p < 0.05$; **$p < 0.01$; ***$p < 0.001$; one-tailed, two-sample t-test.

Supplementary Table 1: Summary of clinico-pathological parameters and results of RhoA and RhoC protein expression in tissue specimens

CaseID	Tissue Specimen	pT	pN	Grading	RhoC (cytoplasmic)	RhoC (nuclear)	RhoA
**1**	NE	-	-	-	1	2	0
**1**	DCIS	-	-	high	1	1	0
**1**	BC	1b	0	G2	1	1	1
**2**	NE	-	-	-	1	0	0
**2**	DCIS	-	-	low	1	0	0
**2**	BC	1b	0	G1	1	0	0
**3**	NE	-	-	-	1	1	0
**3**	DCIS	-	-	low	1	1	0
**3**	BC	1c	0	G1	1	1	0
**4**	NE	-	-	-	1	0	0
**4**	DCIS	-	-	low	1	0	0
**4**	BC	is	x	G1	1	0	0
**5**	NE	-	-	-	1	0	0
**5**	DCIS	-	-	high	1	0	0
**5**	BC	1b	0	G2	1	1	2
**6**	NE	-	-	-	1	0	0
**6**	DCIS	-	-	low	1	0	0
**6**	BC	1c	1a	G1	1	0	0
**7**	NE	-	-	-	1	0	0
**7**	DCIS	-	-	low	1	0	0
**7**	BC	2	0	G1	1	1	0
**8**	NE	-	-	-	1	1	0
**8**	DCIS	-	-	high	1	1	0
**8**	BC	1c	2a	G3	1	1	1
**9**	NE	-	-	-	1	1	0
**9**	DCIS	-	-	high	1	1	2
**9**	BC	1c	1mi	G3	1	0	2

The tables provides pT, pN and grading of breast tissue specimens and results of RhoA and RhoC immunohistochemical analyses. NE= normal epithelium, DCIS=ductal carcinoma in situ and BC = ductal breast carcinoma. RhoC was scored for both cytoplasmic and nuclear expression, RhoA for cytoplasmic expression, with 0 = negative, 1 = weak positivity, 2 = marked positivity.

Supplementary Table 2: List of log fold changes and the false discovery rate-adjusted *p*-values for differential regulation of all genes under RhoA and RhoC induction or CNF1 and CNFY stimulation for days 1 and 7. See Supplementary_Table_2.
